# Quantification of *Pneumocystis jirovecii*: Cross-Platform Comparison of One qPCR Assay with Leading Platforms and Six Master Mixes

**DOI:** 10.3390/jof6010009

**Published:** 2019-12-26

**Authors:** Sarah Dellière, Maud Gits-Muselli, P. Lewis White, Carlo Mengoli, Stéphane Bretagne, Alexandre Alanio

**Affiliations:** 1 Laboratoire de Parasitologie-Mycologie, Groupe Hospitalier Saint-Louis-Lariboisière-Fernand-Widal, Assistance Publique-Hôpitaux de Paris (AP-HP), Université de Paris, 75475 Paris, France; sarah.delliere@gmail.com (S.D.); maud.gits-muselli@pasteur.fr (M.G.-M.); stephane.bretagne@aphp.fr (S.B.); 2 Molecular Mycology Unit, Centre National de la Recherche Scientifique (CNRS), Unité Mixte de Recherche (UMR2000), Institut Pasteur, CEDEX 15, 75724 Paris, France; 3 National Reference Center for Invasive Mycoses and Antifungals (NRCMA), Institut Pasteur, CEDEX 15, 75724 Paris, France; 4 Public Health Wales, Microbiology Cardiff, Heath Park, University Hospital of Wales (UHW), Cardiff CF14 4XW, UK; lewis.white@wales.nhs.uk; 5 Department of Molecular Medicine, University of Padua, 35122 Padua, Italy; mengolicarlo@gmail.com

**Keywords:** pneumocystis, qPCR, diagnosis, standardization, efficiency, threshold, DNA, whole nucleic acid, quantification cycle

## Abstract

Diagnosis of *Pneumocystis jirovecii* pneumonia relies on nucleic acid quantification in respiratory samples. Lack of standardization among molecular assays results in significant differences among assays/centers. To further promote standardization, we compared four thermocyclers and six master mixes for the detection of *P. jirovecii*. Whole nucleic acid (WNA) was extracted from broncho-alveolar lavages. Positive and negative sample extracts were pooled to get enough homogeneous materials. Three master mixes were tested to detect DNA by qPCR (D1, D2, and D3), and three to detect WNA by reverse transcriptase qPCR (W1, W2, and W3) manufactured by Roche, Eurogentec, Applied Biosystem, Invitrogen and Thermofischer Scientific. Experiments were performed on four thermocyclers (Roche LightCycler 480, Qiagen Rotor-Gene Q, Applied Biosystem ABI7500, and QuantStudio). Comparison of quantitative cycle (*Cq*) values between the methods targeting WNA versus DNA showed lower *Cq* values for WNA, independently of thermocycler and master mix. For high and low fungal loads, ∆*Cq* values between DNA and WNA amplification were 6.97 (±2.95) and 5.81 (±3.30), respectively (*p* < 0.0001). Regarding DNA detection, lower *Cq*s were obtained with D1 compared to D2 and D3, with median ∆*Cq* values of 2.6 (*p* = 0.015) and 2.9 (*p* = 0.039) respectively. Regarding WNA detection, no mix was superior to the others. PCR efficiency was not significantly different according to the qPCR platform (*p* = 0.14). This study confirmed the superiority of WNA over DNA detection. A calibration method (e.g., an international standard) for accurate comparative assessment of fungal load seems necessary.

## 1. Introduction

*Pneumocystis jirovecii* is an ascomycetous fungi that thrives in human alveoli and is constantly transmitted among immunocompetent individuals, who are most likely to be the reservoir of the fungus [1]. In immunocompromised patients, *P. jirovecii* causes *Pneumocystis* pneumonia (PCP), mostly in patients with AIDS, hematological malignancies, solid organ transplant, or under immunosuppressive therapy, especially corticosteroids [2,3,4]. In these different populations, the symptoms and therefore the disease severity depend not only on the underlying disease, but also on the fungal load, which is therefore important to quantify [5]. Moreover, *P. jirovecii* is responsible for numerous outbreaks in hospital settings and early detection of carriers with low fungal load and infected patients is essential [6].

Microbiological PCP diagnosis remains a challenge as researchers have struggled to grow *P. jirovecii* in vitro. Thus, diagnosis relies on direct microscopic examination of respiratory samples, detection of 1,3-β-d-glucan (BDG) in serum, and detection of nucleic acid in respiratory samples [5]. The efficiency of microscopy depends on staff experience and false negatives are frequent, particularly when associated with low fungal load in non-HIV patients [7]. BDG shows a great negative predictive value and appears highly sensitive, but is a broad-fungal antigen and frequently yields false positive results not associated with a fungal source [5]. Real-time quantitative PCR (qPCR) is not only more sensitive than microscopy and more specific than BDG, but can also more accurately quantify fungal burden, which is useful for differentiating disease from colonization [8,9].

Over the past 30 years, a number of molecular assays have been developed to detect *P. jirovecii* DNA [5]. Compared to end-point PCR, qPCR is the preferred method for diagnostic laboratories due to the rapid, quantifiable results and lower risk of amplicon contamination associated with this closed-tube format with no post-amplification processing [5,10]. Even if the Minimum information for publication of qPCR experiment (MIQE) guidelines on how best to perform and report qPCR experiments are followed [11], the lack of methodological standardization yields significant differences among diagnostic centers. Indeed, publications describe the use of different DNA targets, probes (hydrolysis probes, hybridization probes, and molecular beacon), reagents, thermocycler equipment, and analysis format.

The requirement for the most sensitive qPCR method is driven by the need for detection of disease or carriage, essential not only for accurately diagnosing PCP, but also for preventing the transition from carriage to PCP and for managing the risk of transmission between susceptible patients [6,12]. In addition, increasing the negative predictive value for a lethal disease in immunocompromised individuals is preferable, particularly when the incidence of disease is low. Indeed, severe fulminant cases with low fungal loads are seen in non-HIV patients, so high sensitivity is paramount [13]. From an infection control perspective, the detection of low fungal loads in patients suspected to only be carriers may require prophylaxis in order to prevent (I) development of PCP in at-risk individuals [14] and (II) transmission and the onset of PCP outbreaks among the immunocompromised population in hospital settings [6].

To allow comparison of quantification for the diagnosis of PCP, studies were recently initiated by the *Pneumocystis* PCR working party of the Fungal PCR Initiative, a study group from the International Society of Human and Animal Mycology (ISHAM) in an attempt to provide a consensus method for *P. jirovecii* qPCR [15]. The first study evaluated the performance variability of 20 in-house and commercial assays in 16 diagnostic centers across Europe, and demonstrated the superiority of reverse transcriptase qPCR (RTqPCR) based on the detection of whole nucleic acid (WNA, detecting both DNA and RNA) over the amplification of DNA alone [15]. In addition, amplification of the target *mtSSU* (mitochondrial small subunit) was associated with lower (earlier) *Cq*s (quantitative cycles) and subsequently better sensitivity in detecting *Pneumocystis* DNA than the other targets—mtLSU (mitochondrial large subunit), MSG (major surface glycoprotein), or tubulin. A second study by the same expert study group comparing 10 different qPCR assays in one center confirmed the first findings [15].

In order to provide further standardization and based on these previous results, the present study compared four different thermocyclers and six master mixes for the PCR diagnosis of PCP. It is known that the performance of other assays, including the detection of the opportunistic fungus *Aspergillus fumigatus*, varies with thermocyclers [16]. Similarly, it has been recently shown that using the same primers, probe, and qPCR amplification protocol for *P. jirovecii* DNA detection still gave a 3-fold variation in *Cq* values when performed in different centers, hypothetically attributed to the kit/enzyme and thermocycler used [15].

## 2. Materials and Methods

### 2.1. Sample Preparation

Residual clinical broncho-alveolar lavage fluids (BALFs) were kept for quality and service evaluation purposes after routine investigations, as permitted by French Health Public Law (CSP Art L1121-1.1). Patients were informed and gave consent for the possibility of using residual material for additional laboratory studies before the BAL procedure.

A total of three *P. jirovecii*-positive BALFs from different genotypes [17] were selected and whole nucleic acid (WNA) was extracted from 900 µL of concentrated BALF pellet (five minutes at 10,000× *g*), including 10 µL per sample of 1:5 diluted internal control (DNA Virus culture, DICD-CY-L100, Diagenode, Seraing, Belgium). Extraction was performed on a Qiasymphony (Qiagen, Hilden, Germany) with the Virus-Pathogen Kit (Qiagen) according to the manufacturer’s instructions. Each whole nucleic acid extract (WNA) was tested separately for inhibitors. WNA were pooled to provide sufficient material for the different equipment and master mix comparisons. A total of 30 *P. jirovecii*-negative BALFs were extracted and pooled to provide a negative control. All aliquots were stored at −20 °C until use. Pooled positive extract was serially diluted using the negative pooled extract to provide 1:5, 1:10, 1:50, 1:100, and 1:1000 dilutions, on the same day that all conditions were tested. Dilutions were performed using the negative pooled extract instead of DNA-free water in order to preserve human WNA concentration.

### 2.2. Evaluation of Different Master Mixes and Thermocyclers

In-house qPCR detecting DNA and RTqPCR detecting WNA targeting the mitochondrial small subunit ribosomal RNA gene (*mtSSU*) (shown to be the most sensitive method) [15] was selected for comparative analysis using different PCR master mixes (*n* = 6) and different thermocycler platforms (*n* = 4), resulting in 24 combinations.

The performance of six different master mixes was comparatively tested. Three were suited for the detection of DNA only: (D1) LightCycler 480 Probes Master (Roche, Mannheim, Germany), (D2) MasterMix Plus Low ROX (Eurogentec, Liège, Belgium), and (D3) Taqman Universal PCR Master Mix (Applied Biosystems, Warrington, UK). Three were suited for the detection of WNA: (W4) Superscript III One step RT-PCR (Invitrogen, Carlsbad, CA, USA), (W5) TaqMan™ Fast Virus 1-Step (Thermo Fischer Scientific, Vilnius, Lithuania), and (W6) LightCycler Multiplex RNA Virus Master (Roche). All mixes, including buffer and enzyme, were used according to the manufacturer’s instructions with a final concentration of 0.3 µM of each primer (Forward 5′-TCATGACCCTTATGAAGTGGGC-3′ and Reverse 5′-GCTCCGACTTCCATCATTGC-3′) and 0.1 µM of probe (5′-FAM-ACGTGCTGCAAAATTTTCTACAATGGG-BHQ1-3′), using molecular-grade nucleic-acid-free water to provide a final reaction volume of 25 µL, which included 5 µL of WNA extract [18]. For the detection of DNA, one activation step at 95 °C for 10 min (as recommended by all manufacturers of the tested kits) was followed by 50 cycles of denaturation at 95 °C for 15 s and annealing at 60 °C for 30 s. For the detection of WNA, the amplification consisted of one step of reverse transcription at 50 °C for 15 min, followed by qPCR with one activation step at 95 °C for two minutes and 50 cycles of denaturation at 95 °C for 15 s and annealing at 60 °C for 30 s. This protocol was adapted in order to fit all protocols recommended by tested kits’ manufacturers, considering that our amplicon was short (76 base pairs) (Table S1) [18].

The performance of four different thermocycler platforms was comparatively tested: a Light Cycler 480 thermocycler (LC480-II; Roche Diagnostics, Rotkreuz, Switzerland), a QuantStudio 7 Flex (ThermoFisher Scientific, Vilnius, Lithuania), an ABI 7500 (Applied Biosystem, Singapore), and a Rotor-Gene Q (Qiagen, Hilden, Germany). Quantification cycle (*Cq*) values were determined with both the second derivative and fit point method for the LC480-II, and only the fit point method for the ABI 7500 and QuantStudio 7 Flex. For the Rotor-Gene Q, the second derivative was used to calculate take-off point. *Cq* was then calculated by linear interpolation. Fit point method was not available with this PCR platform.

### 2.3. Statistical Analysis

Each dilution was tested in duplicate by each piece of equipment and with each master mix. Each replicate was analyzed as an independent result. Negative results were allocated a *Cq* of 45. PCR efficiency was calculated using the formula *e* = 10^−1^/s, where *s* corresponds to the slope of the standard curve, with *Cq* plotted on the y axis and the fungal burden plotted using a common logarithmic scale on the x axis. Analytical sensitivity was evaluated using the *Cq* value obtained and the ability to detect DNA or WNA at the lowest (1:1000) concentration. Graphic and basic statistical analysis including calculation of means, SDs, and *p*-values was performed using Microsoft Excel 2013 and GraphPad Prism 6.0. For the specific results shown in Table 3, the effects of the PCR platforms on *Cq* values are reported as pairwise contrasts (mean differences) between platforms at each target load.

## 3. Results

**Figure 1:**
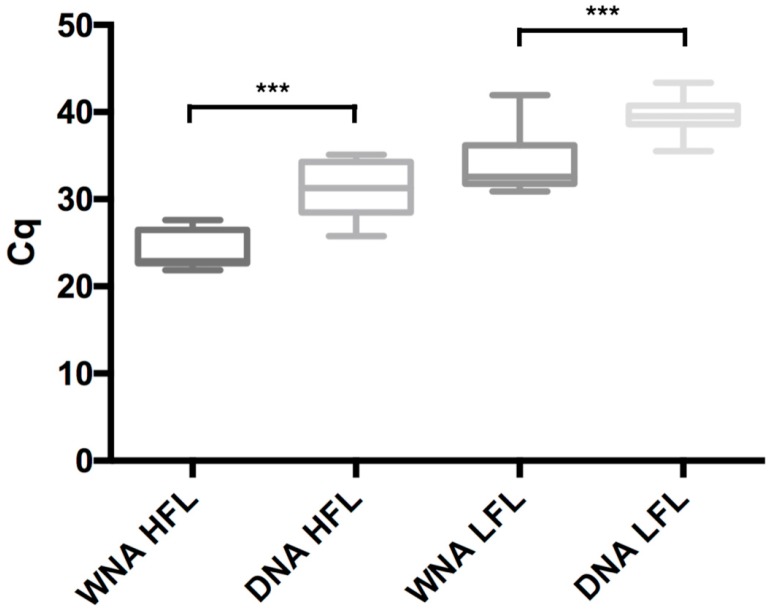
Comparison of quantitative cycle (*Cq*) values independently of mixes and thermocyclers between qPCR method targeting WNA or DNA for high (pure extract, HFL) and low (1:1000 dilution, LFL) fungal load. WNA: whole nucleic acid; *Cq*: quantitative cycle. *** *p* < 0.0001.

Using a single primer pair and probe targeting the *mtSSU* ribosomal RNA gene, a total of six master mixes were tested on four different qPCR platforms for the detection of six different dilutions of PCP-positive nucleic acid extract and one negative control, resulting in 336 tests in duplicate. A comparison of *Cq* values between the RTqPCR method targeting WNA and the qPCR method targeting DNA showed significantly lower *Cq* values for WNA independently of qPCR thermocyclers and master mixes (Figure 1). For the high fungal load (pure extract, HFL), the mean *Cq* difference was 6.97 (±2.95) (*p* < 0.0001) between WNA and DNA. For the low fungal load (1:1000 dilution, LFL), the mean *Cq* difference was 5.81 (±3.30) (*p* < 0.0001) between WNA and DNA. Comparisons of *Cq* values between the two methods for other dilutions were similar (Figure S1).

Master mix performances independent of thermocyclers are shown in Table 1. With mixes designed for DNA detection, lower *Cq* values were obtained with Mix D1 compare to Mixes D2 and D3, with median ∆*Cq* values of 2.6 (*p* = 0.015) and 2.9 (*p* = 0.039) with high fungal load (pure extract), respectively. D1 gave a lower *Cq* value independently of the fungal burden (Table S2). Mean efficiency of PCR reaction was not calculated for DNA detection because *Cq* ranges were too low to obtain an accurate standard curve. In contrast, lower *Cq* values obtained with the same WNA extracts allowed reliable and analyzable standard curves to be obtained. Regarding mixes suitable for WNA detection, no mix appeared superior to the others. Mean efficiencies of RTqPCR reaction were similar (Table 2).

The effects of the PCR platforms on the signal intensity (*Cq*) were previously analyzed by the *Pneumocystis* PCR working party initiative, comparing 20 assays in multiple centers. The analysis of these data showed that Rotor-Gene Q was significantly associated with lower *Cq* values compared to ABI7500 (*p* = 0.028) or LightCycler 480 (*p* = 0.027), regardless of gene targeted (Table 3). In our current study, mean efficiency was not significantly different among qPCR equipment when detecting WNA (*p* = 0.14). Despite similar PCR efficiency, comparing WNA *Cq* values for high fungal loads across the platforms and independently from the master mixes gave mean *Cq* values of 22.6 (±0.2), 22.6 (±0.2), 23.2 (±0.7) and 27.4 (±0.1) with the Rotor-Gene Q, QuantStudio, LC480, and ABI7500, respectively (*p* < 0.0001, one-way ANOVA). Regarding DNA detection, as mentioned previously, accurate PCR efficiency could not be calculated, but the mean *Cq* values for high fungal loads independent from master-mixes were 27.7 (±1.5), 29.2 (±2.2), 33.3 (±1.6), and 33.5 (±1.7) for Rotor-Gene Q, LC480, ABI7500, and QuantStudio, respectively.

Fit point and derivative method for *Cq* calculations were compared on the LC480. The fit point *Cq* determination method gave significantly lower *Cq* values than the second derivative *Cq* determination method with a mean difference of 1.2 (±0.5) *Cq* (*p* < 0.0001). Mean PCR efficiencies for the fit point and the derivative method were 104.4 (±3.6%) and 137.3 (±23.7%), respectively. In summary, the optimal results were obtained when amplifying WNA, with no particular superiority of any enzyme/kit on Rotor-Gene Q, QuantStudio or LC480.

## 4. Discussion

This study compared six master mixes and four qPCR platforms, and confirmed the importance of considering nucleic acid amplification variability and platforms when comparing analytical properties of PCR assays. Comparison of WNA versus DNA amplification showed the increased sensitivity of WNA, with an earlier detection of fungal nucleic acids by 7.0 *Cq* for high fungal loads. As previously suggested [15], our study showed differences according to the amplification mix used. Using the mitochondrial multicopy gene *mtSSU*, previously described as the most sensitive target for the detection of *P. jirovecii* [15], one mix targeting DNA alone (D1) showed a mean difference in *Cq* values up to 2.9-fold *Cq* decrease over two others (D2 and D3). Furthermore, when comparing platforms, the ABI7500 showed a 5-fold *Cq* increase compared to the other platforms, confirming that intrinsic properties of the thermocycler, including detector sensitivity and settings, may significantly modify qPCR results.

The reasons for the better sensitivity of some assays over others are not straightforward. For instance, the advantage of one mix (D1) over the other two was observed only when using DNA, and no mix was superior to the other when using WNA with RTqPCR. Similarly, the QuantStudio platform showed a lower sensitivity than other platforms only when considering DNA, and not for amplification of WNA. These discrepancies according to the type of nucleic acid detected could simply be because of a higher homogeneity of the RTqPCR enzymes/protocols compared with the DNA qPCR enzymes protocols, leading to less variation. The unknown nature of the enzyme and buffer used by the manufacturer prevent further hypothesis. Whatever the possible reasons, it appears that WNA is superior to DNA, both in providing a higher sensitivity and in providing more homogenous results.

Overall, Rotor-Gene Q showed the lowest mean *Cq* values for both DNA and WNA detection, independently of master mixes. This could be attributed to the optimal temperature uniformity of ±0.01 °C over all samples, thanks to its air cooling and centrifugation format [19]. The inconvenience of such a device is the use of Rotor-Discs instead of a 96 well plate, requiring specific additives to work efficiently. One other main difference of these platforms is the light source. QuantStudio and ABI7500 utilize a halogen lamp, giving a broad spectrum light source, which provides the widest choice of detectable fluorophores when paired with appropriate filters. However, this type of lamp decays over time and needs replacement, compared to the light-emitting diodes (LEDs) used by Rotor-Gene Q and LC480. LEDs emit light of a very narrowed wavelength range, and multiple LEDs need to be used to get around this limitation [19].

One must acknowledge some technical limitations when working with *Pneumocystis*. Because the culture of *P. jirovecii* is difficult, there is a reliance on patient samples for quality control purposes and as research material. Only residual samples from routine diagnostic testing are available, which are limited in term of number and volume. Thus, despite pooling positive samples, centrifuged beforehand in order to increase DNA concentration, fungal loads were too low to provide an adequate range of dilution to calculate standard curve and PCR efficiency with the method detecting DNA only according to the MIQE guidelines, which recommend a range of 5-log dilution in the detectable *Cq* range [11]. Other variability factors could not be assessed in this study, such as the possible impact of different WNA or DNA extraction protocols, because only one method was used. This should be addressed in further studies, as noted previously [20,21].

A further objective should be the development of a calibration method (e.g., an international PCP PCR standard) for accurate comparative assessment of fungal load. A synthetic control, such as a plasmid, added in known quantities could be compared to a single copy gene of *P. jirovecii* and thus provide an absolute quantification of the number of organism per unit of respiratory sample. However, such a protocol targeting a single copy gene would be less sensitive than qPCR targeting a multicopy gene (e.g., *mtSSU*) on WNA.

## 5. Conclusions

This study confirmed the impact of qPCR platform and amplification kit/enzyme on *P. jirovecii* nucleic acid quantification. According to our results, the detection of WNA should be preferred with any of the kits/enzymes tested (Superscript III One step RT-PCR (Invitrogen), TaqMan™ Fast Virus 1-Step (Thermo Fischer Scientific), or LightCycler Multiplex RNA Virus Master (Roche)) using either Rotor-Gene Q, LC480, or QuantStudio. To date, no consensual threshold value exists to distinguish between high (infection) and low (carriage) fungal loads in respiratory samples, and this distinction mostly relies on the center’s expertise and experience with their own assay/qPCR method. It will not be possible to derive a single threshold if performance varies across platforms and with different master mixes. Understanding variability factors is the first step toward a standardized method validated by health care agencies. RTqPCR (amplifying both RNA and DNA) targeting the *mtSSU* gene appears to be the most sensitive assay, is less affected by variations in master mix and amplification platform, and could be considered the best screening test for detecting *P. jirovecii* nucleic acid. However, variability observed in this study due to master mix and thermocycler prevents the application of a consensual threshold until a definite method has been determined.

## Figures and Tables

**Table 1:** Analysis of mixes’ performance independently of qPCR equipment. Mean efficiency of PCR reaction could not be calculated for the DNA method as *Cq* ranges were too low. HFL: high fungal load (pure extract); LFL: low fungal load (1:1000); *Cq*: quantitative cycle. (D1) LightCycler 480 Probes Master, (D2) MasterMix Plus Low ROX, (D3) Taqman Universal PCR Master Mix, (W4) Superscript III One step RT-PCR, (W5) TaqMan™ Fast Virus 1-Step, (W6) LightCycler Multiplex RNA Virus Master.

Mix		Mean *Cq* HFL (±SD)	*p*	Mean *Cq* LFL (±SD)	*p*	Mean PCR Efficiency in % (±SD)
DNA detection	Mix D1	28.8 (±2.7)	0.009	37.2 (±2.1)	0.008	nd
	Mix D2	31.6 (±3.4)		40.0 (±2.2)		nd
	Mix D3	30.7 (±4.7)		39.5 (±2.4)		nd
WNA detection	Mix W4	24.0 (±2.2)	NS	33.3 (±2.8)	NS	104.5 (±7.4)
	Mix W5	23.5 (±2.4)		33.4 (±3.0)		100.5 (±2.9)
	Mix W6	24.4 (±2.2)		33.7 (±3.6)		103.8 (±9.3)

NS: not significant; SD: standard deviation; nd: Not done.

**Table 2:** RT-qPCR efficiency (%) according to platform and master mixes. (Mix 4) Superscript III One step RT-PCR, (Mix 5) TaqMan^TM^ Fast Virus 1-Step, (Mix 6) LightCycler Multiplex RNA Virus Master.

Mix	ABI7500	QuantStudio	Rotor-Gene Q	LC480	
	Fit Point	Fit Point	Fit Point	Fit Point	Derivative
Mix W4	99.8	100.1	115.4	102.5	110.0
Mix W5	96.2	102.4	101.3	102.1	152.7
Mix W6	92.1	113.5	101.1	108.6	149.3
Mean gap from perfect efficiency (i.e., 100%) (±SD)	4.0 (±3.8)	5.3 (±7.1)	5.9 (±8.1)	4.4 (±3.6)	37.3 (±23.7)

SD: standard deviation.

**Table 3:** Effect of the thermocycler platform on the *Cq* values of three panel specimens (1:1, 1:100, and 1:1000 dilutions) tested multicentrically (20 assays across 16 international laboratories) [15]. Only significant (*p* < 0.05) and quasi-significant (0.05 < *p* < 0.1) results are shown.

Target Load	Platform 1	Platform 2	Contrast	Std. Err.	*p*	Better
1:1000	none	none	-	-	-	-
1:100	Quantstudio	ABI 7500	−5.537	2.858	0.053	first
	Rotor-Gene Q	ABI 7500	−4.080	1.853	0.028	first
1:1	Light Cycler 480	Quantstudio	4.523	2.490	0.069	second
	Rotor-Gene Q	Light Cycler 480	−3.378	1.525	0.027	first

## Supplementary Materials

The following are available online at https://www.mdpi.com/2309-608X/6/1/9/s1.
