# Supplementary material for: Quantification of Pneumocystis jirovecii: Cross-Platform Comparison of One qPCR Assay with Leading Platforms and Six Master Mixes
Source: J Fungi (Basel). 2019 Dec 26;6(1):9. doi: 10.3390/jof6010009 (PMC7151141; doi:10.3390/jof6010009)
Supplement: Supplementary file 1 [file jof-06-00009-s001.pdf]

Journal of
Fungi

MDPI

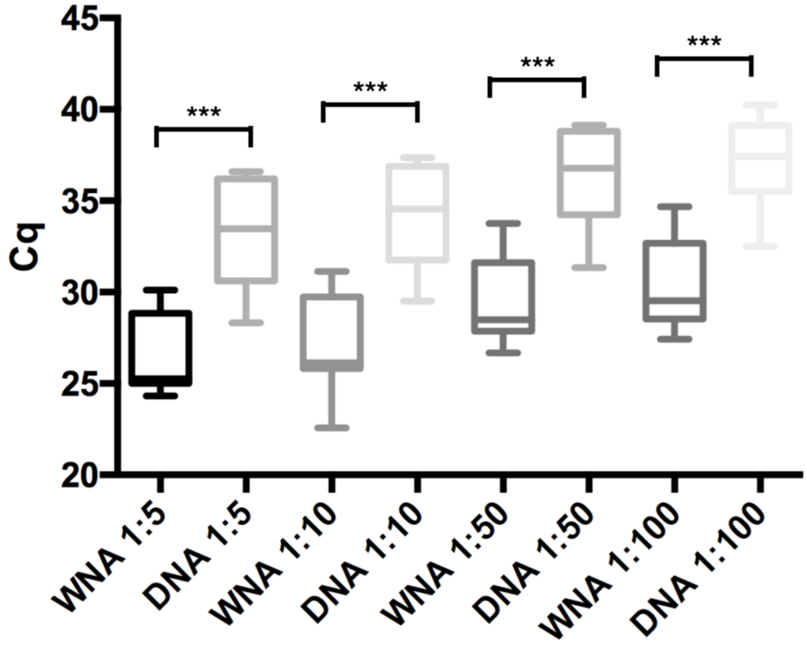

Figure S1. Comparison of Cq values independently of mixes and thermocyclers between qPCR method targeting WNA or DNA for 1:5, 1:10, 1:50 and 1:100 dilutions. WNA: whole nucleic acid; Cq: quantitative cycle. *** p<0.0001.

Table S1. PCR protocol used for RT-qPCR and recommended by manufacturers of (W4) Superscript III One step RT-PCR, (W5) TaqMan™ Fast Virus 1-Step, (W6) LightCycler Multiplex RNA Virus Master respectively.

	RT	RT inactivation / DNA polymerase activation	denaturation	annealing
Protocol used	50 °C for 15 min	95 °C for 2 min	95 °C for 15 sec	60 °C for 30 sec
W4	50 °C for 15 min	95 °C for 2 min	95 °C for 15 sec	60 °C for 30 sec
W5	50 °C for 15 min	95 °C for 20 sec	95 °C for 15 sec	60 °C for 60 sec
W6	50 °C for 15 min	95 °C for 30 sec	95 °C for 5 sec	60 °C for 30 sec

Table S2. Analysis of mixes performance independently from qPCR equipment at 1:5, 1:10, 1:50 and 1:100 dilutions. Cq: quantitative cycle. (D1) LightCycler 480 Probes Master, (D2) MasterMix Plus Low ROX, (D3) Taqman Universal PCR Master Mix, (W4) Superscript III One step RT-PCR, (W5) TaqMan™ Fast Virus 1-Step, (W6) LightCycler Multiplex RNA Virus Master.

		1:5	*P*	1:10	*P*	1:50	P	1:100	P
DNA detection	D1	31.5 (± 2.2)	0.005	32.6 (± 2.2)	0.006	34.9 (± 2.0)	0.0 07	35.8 (± 1.9)	0.01
	D2	34.4 (± 2.1)		35.3 (± 2.1)		37.4 (± 1.8)		38.1 (± 1.1)	
	D3	33.3 (± 3.6)		34.2 (± 3.4)		36.4 (± 3.4)		37.1 (± 3.0)	
WNA detection	W4	26.5 (± 2.2)	NS	27.5 (± 2.3)	NS	29.7 (± 2.3)	NS	30.6 (± 2.5)	NS
	W5	26.1 (± 2.2)		26.5 (± 2.7)		29.0 (± 2.2)		28.8 (± 2.3)	
	W6	26.5 (± 2.3)		27.1 (± 2.6)		29.5 (± 2.6)		30.5 (± 2.6)	

NS: not significant; SD: standard deviation; nd: Not done.
